# Cancerous Inhibitor of Protein Phosphatase 2A Mediates Bortezomib-Induced Autophagy in Hepatocellular Carcinoma Independent of Proteasome

**DOI:** 10.1371/journal.pone.0055705

**Published:** 2013-02-01

**Authors:** Hui-Chuan Yu, Duen-Ren Hou, Chun-Yu Liu, Chen-Si Lin, Chung-Wai Shiau, Ann-Lii Cheng, Kuen-Feng Chen

**Affiliations:** 1 Department of Medical Research, National Taiwan University Hospital, Taipei, Taiwan; 2 National Center of Excellence for Clinical Trial and Research, National Taiwan University Hospital, Taipei, Taiwan; 3 School of Veterinary Medicine, National Taiwan University, Taipei, Taiwan; 4 Department of Oncology, National Taiwan University Hospital, Taipei, Taiwan; 5 Department of Chemistry, National Central University, Taoyuan, Taiwan; 6 Division of Hematology and Oncology, Department of Medicine, Taipei Veterans General Hospital, Taipei, Taiwan; 7 Institute of Biopharmaceutical Sciences, National Yang-Ming University, Taipei, Taiwan; St. Luc University Hospital, Belgium

## Abstract

Previously, we reported that cancerous inhibitor of protein phosphatase 2A (CIP2A) mediates the apoptotic effect of bortezomib in hepatocellular carcinoma (HCC). Here, we report a proteasome-independent mechanism by which bortezomib induces autophagy in HCC. Our data indicate that bortezomib activated autophagy in a dose- and time- dependent manner in HCC cell lines including Huh-7, Sk-Hep1, and Hep3B. Bortezomib downregulated CIP2A, phospho-Akt (P-Akt) and phospho-4EBP1 (P-4EBP1) in a dose- and time-dependent manner in all tested HCC cells. Ectopic expression of CIP2A abolished the effect of bortezomib on autophagy. Co-treatment of bortezomib and calyculin A, a PP2A inhibitor, reduced the effect of bortezomib on P-Akt, P-4EBP1, and autophagy. Increased phosphorylation of either Akt or 4EBP1 by ectopic overexpression protected cells from bortezomib-induced autophagy. Furthermore, we examined the effect of ΔBtz, a bortezomib derivative that closely resembles bortezomib structurally but has no proteasome activity, in HCC. Interestingly, ΔBtz demonstrated similar effects to bortezomib on autophagy, CIP2A, P-Akt and P-4EBP1, suggesting that the effect of bortezomib on autophagy is independent of proteasome inhibition. Moreover, our *in vivo* data showed that both bortezomib and ΔBtz inhibited tumor growth, downregulated CIP2A, P-Akt and induced autophagy in Huh-7 tumors. In conclusion, bortezomib induces autophagy in HCC through a CIP2A-PP2A-Akt-4EBP1 pathway.

## Introduction

Hepatocellular carcinoma (HCC) is the fifth most common solid tumor worldwide [1]. Advanced HCC is characterized by frequent resistance to conventional chemotherapeutic agents and radiation. There is thus clearly a need to develop new therapeutic targets and strategies for HCC therapy. Recently, the autophagy pathway has emerged as a promising new target in cancer treatment. Autophagy is known as a homeostatic mechanism for maintaining cellular integrity and is a catabolic process that involves degradation of cytoplasmic components via the lysosomal machinery [2]. Autophagy plays multiple roles in cancer: it may promote cancer cell death or survival depending on the complex interactions among metabolic stress, pathways of apoptosis and autophagy [3], [4], [5]; and perturbation of autophagy can also contribute to tumorigenesis [3], [6]. A better understanding of autophagy regulation may facilitate discovery of new potential therapeutic targets in HCC.

Bortezomib is the first in-class dipeptide boronate proteasome inhibitor specifically designated to target the 26S proteasome [7], [8]. Bortezomib has been approved for treatment of multiple myeloma and mantle cell non-Hodgkin's lymphoma (NHL) and is under clinical investigation for use in other cancers [9], [10]. In addition to its effects on apoptosis induction, cell-cycle inhibition (G2-M phase arrest) and many other cellular mechanisms associated with proteasome inhibition [10], [11], [12], bortezomib has recently been shown to induce autophagy in hypoxic HeLa cervical carcinoma cells in response to activated endoplasmic reticulum (ER) stress [13], in human prostate cancer cells through EIF-2α phosphorylation [14], and in human head and neck squamous cell carcinoma cells in association with proteasome-dependent JNK activation and Bcl-2 phosphorylation [15]. Although these studies commonly suggest bortezomib-induced autophagy correlates with its proteasome inhibition [13], [14], [15], the exact mechanism of bortezomib-induced autophagy is not fully understood.

It is well known that mammalian target of rapamycin (mTOR) is a key regulator of cell growth and autophagy [16]. Furthermore, activated mTOR complex 1 (mTORC1), one of the two major mTOR components, activates S6K and phosphorylates 4EBP-1 (thereby releasing 4EBP-1 from eIF4E) promoting mRNA translation [17]. In addition, mTORC1 directly interacts with and inhibits the ULK1 complex, an essential component in autophagy initiation [18]. Mediation of growth factor signaling by mTOR is primarily in response to the phosphatidylinositol 3-kinase (PI3K)/Akt pathway [19]. Akt has a crucial role in cancer cell survival and apoptosis regulation, and recent studies have shown that inhibition of Akt also promotes autophagy [20], [21], [22]. In HCC, the Akt pathway has been shown to be constitutively activated and correlated with a worse prognosis [23]. Our previous study also demonstrated that downregulation of p-Akt is a major molecular determinant of bortezomib-induced apoptosis in HCC cells [24]. It is noteworthy that negative regulation of Akt signaling can be achieved by phosphatases, such as phosphatase and tensin homologue deleted on chromosome ten (PTEN) and protein phosphatase 2A (PP2A). PTEN is a dual protein/lipid phosphatase that counteracts PI3K/Akt signaling by dephosphorylating phosphatidylinositol-3,4,5-trisphosphate (PIP3) at the 3-position [20]. In contrast, PP2A is a serine/threonine protein phosphatase that can directly dephosphorylate p-Akt and p-ERK [25]. PP2A is composed of catalytic C subunit (PP2Ac), scaffolding A subunit (PR65) and regulatory B subunits [26]. PP2A has been suggested to be a tumor suppressor [27]. For example, increased PP2A activity can induce apoptosis through inactivation of Bcl-2 or activation of Bad [28]. PP2A also regulates the cell cycle, cell survival and proliferation by either directly or indirectly inhibiting cdc2, MAPK and Akt kinases [28]. Our recent data also indicated that bortezomib enhances PP2A activity thereby downregulating p-Akt and inducing apoptosis in HCC cells [29]. Moreover, several cellular inhibitors of PP2A, such as SET [30] and CIP2A have been identified [31]. CIP2A has emerged as a novel oncoprotein and a growing number of reports show that it is overexpressed in many human malignancies, including HCC [32], [33], [34], [35], [36], [37], [38], [39]. CIP2A has been shown to stabilize c-Myc oncoprotein by inhibiting PP2A activity toward c-Myc, thus promoting anchorage-independent cell growth and *in vivo* tumor formation [31]. Recently, we further demonstrated that CIP2A, through inhibition of PP2A-dependent p-Akt inactivation, mediates the apoptotic effect of bortezomib in HCC cells [40].

In this study, we report a proteasome-independent mechanism by which bortezomib induces autophagy in HCC. Our present work suggests that bortezomib induces autophagy in HCC through a CIP2A-PP2A-Akt-4EBP1 pathway.

## Materials and Methods

### Reagents and antibodies

Bortezomib (Velcade®) was kindly provided by Millennium Pharmaceuticals. For *in vitro* studies, bortezomib at various concentrations was dissolved in DMSO and then added to cells in Dulbecco's modified Eagle's medium (DMEM) containing 5% fetal bovine serum (FBS). The final DMSO concentration was 0.1% after addition to medium. Calyculin A and 3-Methyladenine (3-MA) were purchased from Cayman Chemical (Ann Arbor, MI). Antibodies for immunoblotting such as anti-Akt1, were purchased from Santa Cruz Biotechnology (San Diego, CA). Other antibodies such as anti-LC3, -P-Akt (Ser473), -4EBP1, -P-4EBP1, -P-mTOR, -mTOR, -P-S6K, -S6K, anti-S6, -P-S6, -NF-κB, -IκB-α, -ATG3, -ATG5, -ATG7, and -Beclin 1 were from Cell Signaling (Danvers, MA).

### Cell culture and western blot analysis

The Sk-Hep1 and Hep3B cell lines were obtained from American Type Culture Collection (Manassas, VA). The Huh-7 HCC cell line was obtained from the Health Science Research Resources Bank (Osaka, Japan; JCRB0403). Cells were maintained in DMEM supplemented with 10% FBS, 100 units/mL, penicillin G, 100 µg/mL streptomycin sulfate, and 25 µg/mL amphotericin B in a 37°C humidified incubator and an atmosphere of 5% CO_2_ in air. western blot analysis was performed as previously reported.

### Autophagy analysis

Drug-induced autophagy was assessed by several assays including: (1) Western blot analysis of microtubule-associated protein 1 light chain 3 (LC3-II) as described previously; (2) Immunofluorescence of LC3-II. Briefly, Huh7 cells were seeded in a 6-cm dish. After being washed with PBS, cells were treated with 800 nM bortezomib for 16 hours, and fixed with ice-cold 4% paraformaldehyde. The fixed cells were subsequently incubated with the primary antibody rabbit anti-LC3II (1∶200, #3868, Cell Signaling Technology, Danvers, MA), in blocking solution (1% bovine serum albumin in TBST) for 1 hour at room temperature and then stained with anti-rabbit IgG (H+L), F(ab′)2 Fragment (Alexa Fluor® 488 Conjugate, #4412, Cell Signaling) and DAPI. Cells were examined under a LEICA DM2500 fluorescence microscope; (3) Flow cytometry analysis of LC**3**-II. Briefly, Huh7 and Hep3B were seeded in a 6-cm dish. After being washed with PBS, cells were treated with 800 nM bortezomib for 16 and 24 hours, fixed with ice-cold 4% paraformaldehyde, and then treated with ice-cold 100% methanol. The fixed cells were subsequently incubated with the primary antibody rabbit anti-LC3II (1∶100, #3868, Cell Signaling) in blocking solution (1% bovine serum albumin in TBST) for 1 hour at room temperature, and then stained with anti-rabbit IgG (H+L), F(ab′)2 Fragment (Alexa Fluor® 488 Conjugate, #4412).

### Immunofluoresence of LC3-GFP and acridine orange

Huh7 cells, grown on coverslips, were transfected with EGFP-LC3 plasmid, followed by 400 nM bortezomib treatment for 24 hours. The cells were then rapidly washed with PBS and fixed at room temperature for 15 minutes with 4% paraformaldehyde. After being washed with PBS twice, the cells were blocked with 1% bovine serum albumin in TTBS and then mounted. The subcellular distribution of EGFP-LC3 was observed under a fluorescence microscope (LEICA DM2500). For acridine orange staining, SK Hep1 cells were seeded in a 6-cm dish. After being washed with PBS, cells were treated with 800 nM bortezomib for 16 hours, fixed with ice-cold 4% paraformaldehyde for 30 minutes at room temperature then stained with acridine orange (5 µg/ml) for 5 minutes at room temperature. Cells were examined under a LEICA DM2500 fluorescence microscope.

### HCC cells with constitutively active CIP2A

CIP2A cDNA (KIAA1524) was purchased from Origene (RC219918; Rockville, MD). Briefly, following transfection, cells were incubated in the presence of G418 (0.78 mg/mL). After 8 weeks of selection, surviving colonies, i.e., those arising from stably transfected cells, were selected and individually amplified. HCC cells with stable expression of CIP2A-myc were then treated with drugs, harvested, and processed for western blot analysis.

### Xenograft tumor growth

Male NCr athymic nude mice (5–7 weeks of age) were obtained from the National Laboratory Animal Center (Taipei, Taiwan). The mice were housed in groups and maintained under standard laboratory conditions on a 12-h light-dark cycle. They were given access to sterilized food and water *ad libitum*. All experimental procedures using these mice were performed in accordance with protocols approved by the Institutional Laboratory Animal Care and Use Committee of National Taiwan University (Permit Number: 20080205). Each mouse was inoculated s.c. in the dorsal flank with 1×10^6^ Huh-7 cells suspended in 0.1 ml of serum-free medium containing 50% Matrigel (BD Biosciences, Bedford, MA). When tumors reached 200–300 mm^3^, mice received an intraperitoneal injection of bortezomib (1 mg/kg body weight) or ΔBtz (1 mg/kg body weight) twice weekly for the duration of treatment. Controls received vehicle.

### Statistical analysis

Tumor growth data points are reported as mean tumor volume ± SE. Comparisons of mean values were performed using the independent samples *t* test in SPSS for Windows 11.5 software (SPSS, Inc., Chicago, IL).

## Results

### Bortezomib induces autophagy in HCC

**Figure 1:**
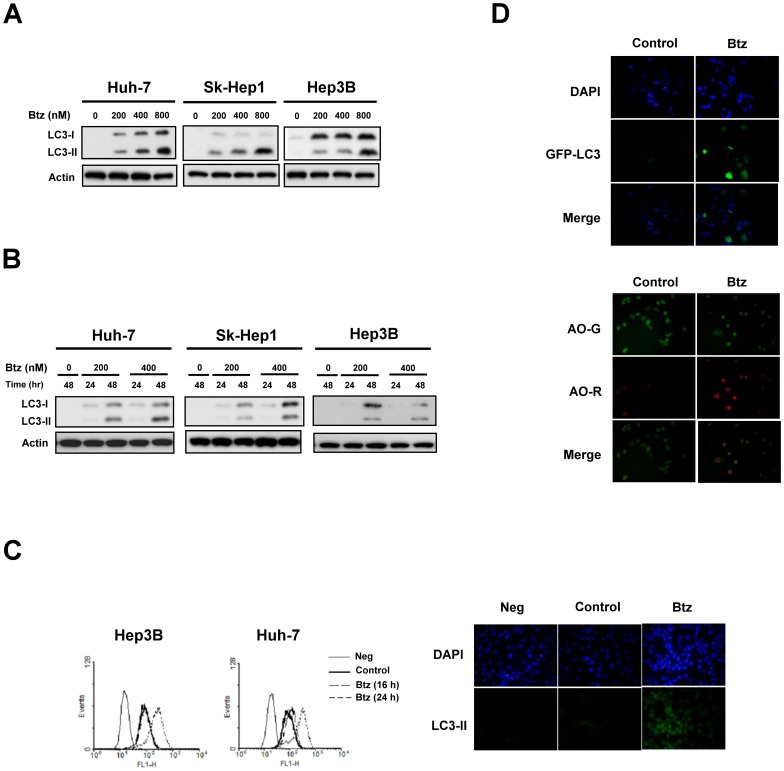
Bortezomib induces autophagy in HCC. A, dose-dependent effects of bortezomib-induced autophagy. HCC cells were treated with bortezomib at the indicated concentrations for 24 hours. Cell lysates were prepared for immunoblotting of microtubule-associated protein 1 light chain 3 (LC3). B, time-dependent effects of bortezomib-induced autophagy. Cells were exposed to bortezomib at the indicated concentrations for 24 or 48 hours. C, effects of bortezomib on LC3-II. *Left,* analysis of LC3-II staining by flow cytometry. Cells were treated with bortezomib at 800 nM for 16 or 24 hours. *Right,* analysis of LC3-II immunofluorescence. Hep3B cells were treated with bortezomib at 800 nM for 24 hours. D, *top,* LC3-GFP staining. *Bottom,* acridine orange stain.

To investigate the effect of bortezomib on autophagy in HCC, we first examined the expression of microtubule-associated protein 1 light chain 3 (LC3-II), a LC3-phosphatidyl-ethanolamine conjugate, that is a hallmark of autophagy. As shown in Fig. 1A, bortezomib induced autophagy in a concentration-dependent manner starting at a concentration of 200 nM in Huh-7, Sk-Hep1 and Hep3B cells, as evidenced by the increase in LC3-II. In addition, we performed a time-dependent analysis of bortezomib-induced autophagy. Our data show that bortezomib upregulated the expression of LC3-II in a time-dependent manner (Fig. 1B). Next, we detected the expression of LC3-II by flow cytometry. As shown in Fig. 1C *left*, bortezomib increased the levels of LC3-II significantly after bortezomib treatment of 24 hours. Moreover, bortezomib-induced autophagy was also confirmed by LC3-II immunofluoresence staining (Fig. 1C *right*). The stimulatory effect of bortezomib on autophagy was confirmed by an increase in the GFP-LC3 dots in the cells treated with the drug, as examined by GFP-LC3 assay (Fig. 1D *top*), and also by an increase in acridine orange staining for acidic vesicular organelles (Fig. 1D *bottom*). These data indicate that bortezomib significantly activates autophagy in HCC.

### Inhibition of CIP2A-Akt-4EBP1 signaling pathway is associated with bortezomib-induced autophagy in HCC

**Figure 2:**
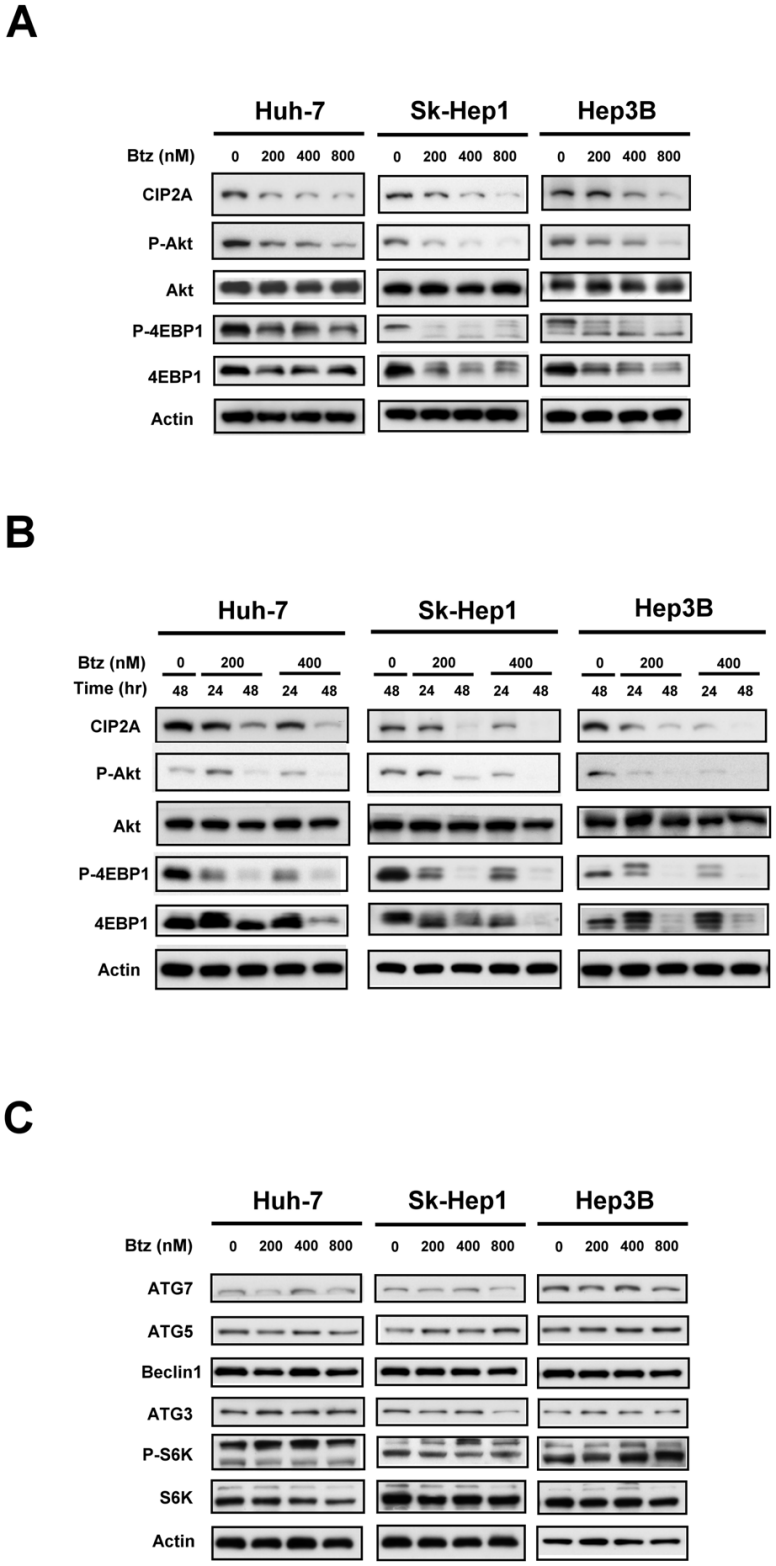
Inhibition of CIP2A-PP2A-Akt-4EBP1 signaling pathway is associated with bortezomib-induced autophagy in HCC. A, dose-dependent analysis of CIP2A, Akt and 4EBP1 in HCC cells. Cells were exposed to bortezomib at the indicated concentrations for 24 hours. B, time-dependent analysis of CIP2A, Akt and 4EBP1 in HCC cells. Cells were exposed to bortezomib at the indicated concentrations for 24 or 48 hours. C, dose-dependent analysis of autophagy-related proteins. Cells were exposed to bortezomib at the indicated concentrations for 24 hours.

Our previous work identified that inhibition of CIP2A is the major determinant of bortezomib-induced apoptosis in HCC cells. To further explore the mechanism by which bortezomib induced autophagy in HCC, we next examined whether CIP2A plays a role in bortezomib-induced autophagy in HCC. As shown in Fig. 2A and B, bortezomib downregulated the protein levels of CIP2A, P-Akt, P-4EBP1 and induced autophagy in all HCC cell lines, including Huh-7, Sk-Hep1 and Hep3B, in a concentration- and time- dependent manner. Furthermore, bortezomib did not significantly alter the expression levels of other autophagy-related proteins including ATG7, ATG5, Beclin 1, ATG3, P-S6K, and S6K (Fig. 2C). These results suggest that inhibition of CIP2A-Akt-4EBP1 is associated with bortezomib-induced autophagy in HCC cells.

### Target validation of CIP2A-PP2A-Akt-4EBP1

**Figure 3:**
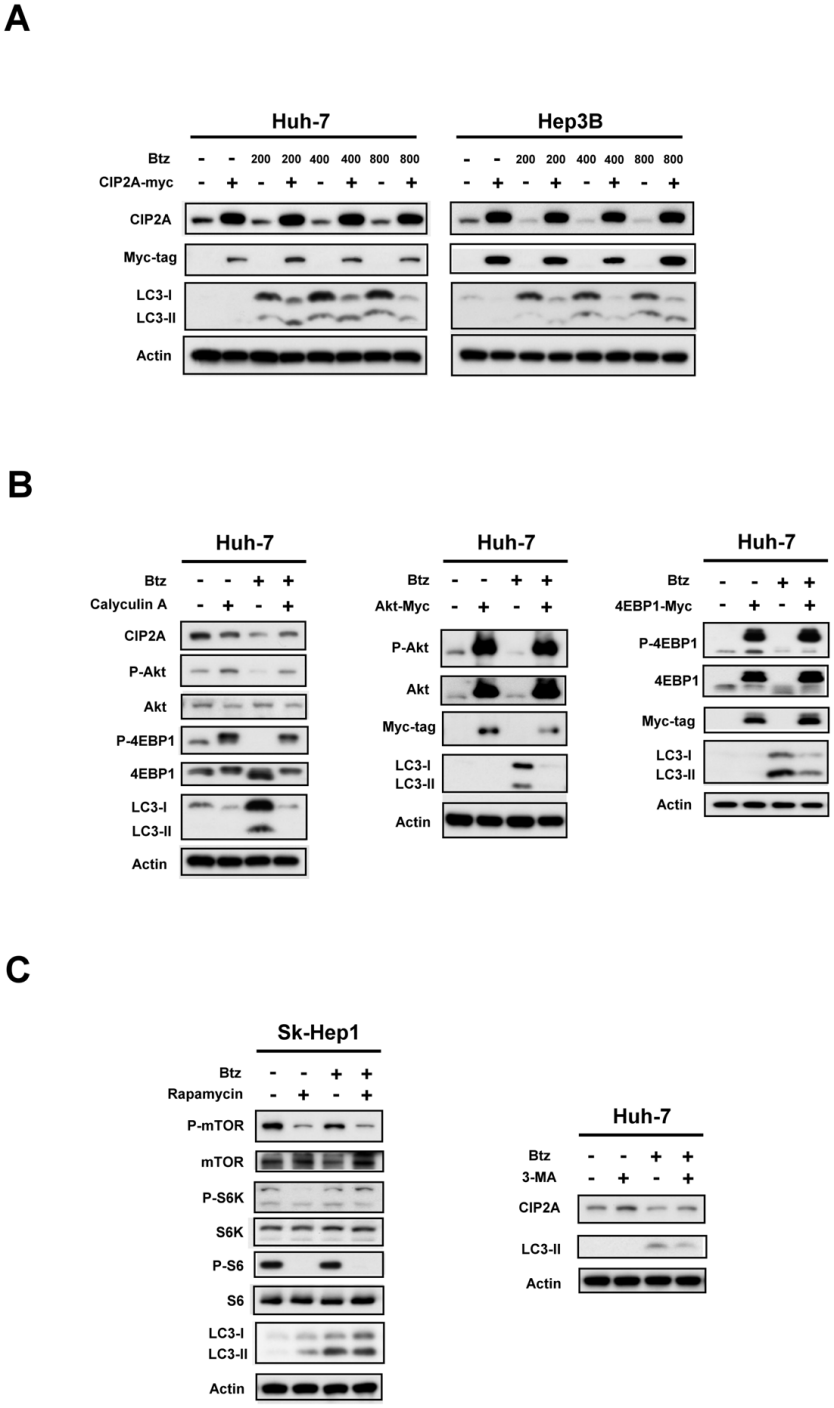
Target validation of CIP2A-Akt-4EBP1. A, ectopic expression of CIP2A-myc abolishes effects of bortezomib on autophagy in Huh-7 and Hep3B cells. Cells were transfected with CIP2A-myc and were selected for 8 weeks by G-418. Analysis of autophagy was performed by western blot (WB) after cells were sequentially exposed to DMSO or bortezomib (200 nM or 400 nM or 800 nM) for 24 hours. B, *left,* co-treatment with calyculin A, a PP2A inhibitor, reverses the effect of bortezomib on CIP2A, P-Akt, P-4EBP1 and autophagy. Cells were treated with calyculin A (1 mM) or bortezomib (800 nM) for 24 hours. *Middle,* ectopic expression of Akt1 reduced the effects of bortezomib on autophagy in Huh-7 cells. *Right,* ectopic expression of 4EBP1 reduced the effects of bortezomib on autophagy in Huh-7 cells. C, *left,* co-treatment with rapamycin, a mTOR inhibitor, enhanced bortezomib-induced autophagy in Huh-7 cells. Cells were treated with rapamycin (100 nM) and/or bortezomib (400 nM) for 24 hours. *Right,* Co-treatment with autophagy inhibitor 3-Methyladenine (3-MA) reduced bortezomib-induced autophagy. Huh7 cells were treated with bortezomib (800 nM) and/or 3-MA (1 mM) for 16 hours.

To validate the role of CIP2A in mediating the effect of bortezomib on autophagy in HCC, we overexpressed CIP2A in HCC. We found that ectopic expression of CIP2A protected cells from bortezomib-induced autophagy in Huh-7 and Hep3B cells, indicating that CIP2A plays a key role in mediating the autophagic effect of bortezomib in HCC cells (Fig. 3A). In addition, adding calyculin A, a PP2A inhibitor, reduced the effect of bortezomib on CIP2A, P-Akt, P-4EBP1 and autophagy in Huh-7 (Fig. 3B *left*). overexpression of Akt1 abolished bortezomib-induced autophagy significantly (Fig. 3B *middle*). Ectopic expression of 4EBP1 also reduced the effect of bortezomib on autophagy in Huh-7. These data indicate that the CIP2A-PP2A-Akt-4EBP1 signaling pathway plays a key role in mediating bortezomib's effect on autophagy in HCC. Furthermore, we examined the effect of a combination of bortezomib and rapamycin, a mTOR inhibitor, on autophagy. Our data showed co-treatment with rapamycin and bortezomib increased autophagy significantly (Fig. 3C *left*). Furthermore, 3-MA, an autophagy inhibitor, reversed the autophagic effect of bortezomib in Huh-7 cells (Fig. 3C *right*). Notably, we have performed all the experiments above (Figure 3) in each HCC cell lines and have found that there was no significant difference between these cell lines with regard to the effect of bortezomib on the validated targets. Only some of the representative data have been shown here.

### The effect of bortezomib on autophagy and CIP2A is independent of the proteasome

**Figure 4:**
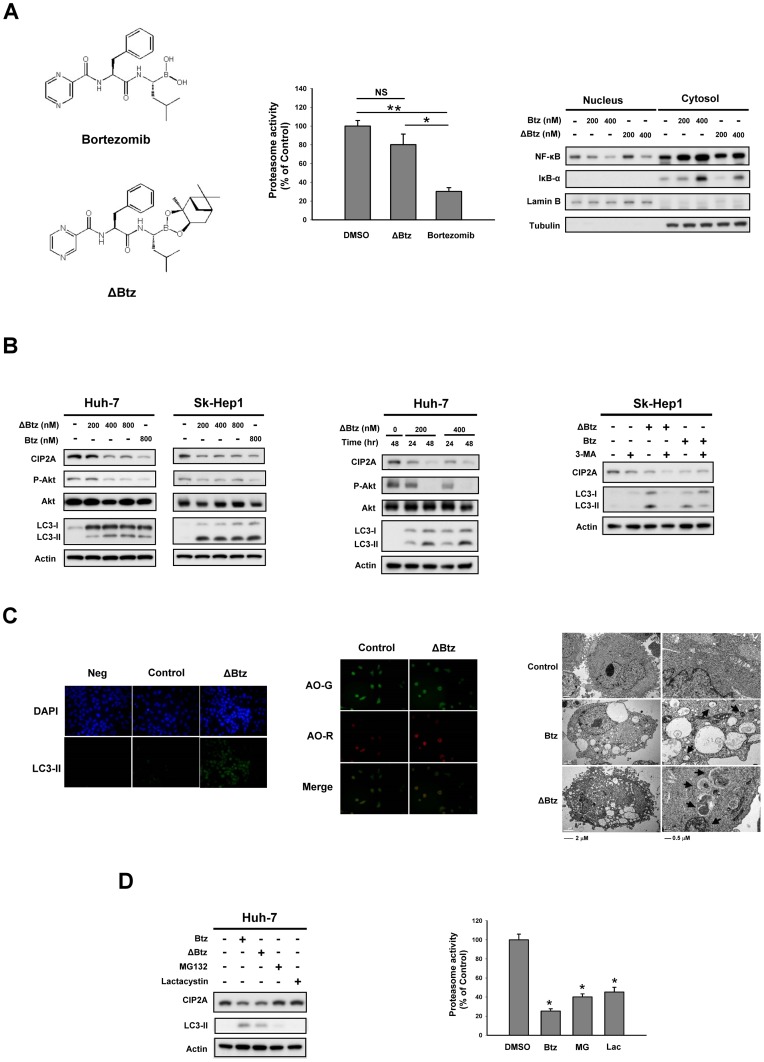
Proteasome-independent effects of bortezomib on autophagy and CIP2A. A, *left,* chemical structures of bortezomib and ΔBtz. *Middle,* effects of bortezomib and ΔBtz on proteasome inhibition. *Right,* effects of bortezomib and ΔBtz on NF-κB. B, *left,* dose-dependent effects of ΔBtz on CIP2A, Akt and LC-3. *Middle,* time-dependent effects of ΔBtz on CIP2A, Akt and LC-3. *Right,* Co-treatment with autophagy inhibitor 3-MA reduced ΔBtz-induced autophagy. C, *left*, effects of ΔBtz on LC3-II immunofluoresence. *Middle*, effects of ΔBtz on acridine orange staining. *Right,* Sk-Hep1 cells treated with bortezomib (800 nM) or ΔBtz (800 nM) or vehicle were harvested, fixed and embedded in spur resin. Seventy nanometer thin sections were cut and examined at 120 Kv with a JEM-1400 transmission electron microscope. Arrows indicate autophagic vacuoles. D, Other proteasome inhibitors did not induce autophagy in HCC. *Left*, effects of MG-132 and lactacystin on CIP2A and LC-3. *Right*, effects of bortezomib, MG-132 and lactacystin on proteasome inhibition.

To examine whether the effect of bortezomib on autophagy and CIP2A is related to its proteasome inhibition activity, we synthesized a bortezomib derivative (ΔBtz), which is similar to bortezomib structurally except that it lacks a boronic acid functional group, a critical contributor to proteasome activity (Fig. 4A *left*). Compared with bortezomib, ΔBtz had little effect on proteasome inhibition (Fig. 4A *middle*) or on NF-κB inhibition (Fig. 4A *right*). NF-κB is a known proteasome inhibitor target. Interestingly, ΔBtz showed significant effects on CIP2A, P-Akt, and P-4EBP1 in a concentration- and time-dependent manner in HCC similar to bortezomib (Fig. 4B *left* & *middle*). Co-treatment with the autophagy inhibitor 3-MA reduced the autophagic effect of ΔBtz (Fig. 4B *right*). ΔBtz increased the expression of LC3-II (Fig. 4C *left*), and also increased acridine orange staining for acidic vesicular organelles (Fig. 4C *middle*). Using an electron microscopy technique, the numbers of autophagic vacuoles were shown to be markedly increased in Sk-Hep1 cells treated with 800 nM bortezomib or ΔBtz, but not in the control (Fig. 4C *right*). Moreover, we examined the effects of other proteasome inhibitors MG132 and lactacystin, on autophagy and CIP2A. Our data showed that neither MG132 nor lactacystin had significant effects on autophagy or CIP2A (Fig. 4D *left*). However, as expected, these two proteasome inhibitors exhibited significant proteasome inhibition activity (Fig. 4D *right*). These data indicate that the effect of bortezomib on autophagy is independent of the proteasome.

### Effect of bortezomib and ΔBtz on HCC xenograft tumor

**Figure 5:**
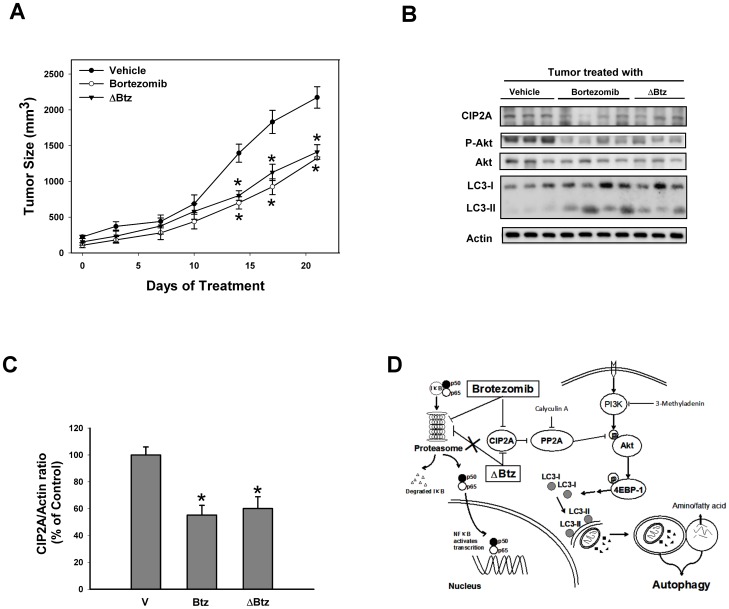
*In vivo* effect of bortezomib and ΔBtz on Huh-7 xeonograft nude mice. A, tumor growth curves of Huh-7. Points, mean (n = 6); bars, SE. *P<0.05; ** *P*<0.01. B, western blot analysis of CIP2A, P-Akt, Akt1 and LC-3 in Huh-7 tumors. C, immunoblots were scanned by a UVP BioSpectrum AC image system and quantitated using VisionWork LS software to determine the ratio of the level of CIP2A to actin. *Columns*, mean; *bars*, SD (n = 3, *P<0.05). D, summary of the mode of actions of bortezomib.

To confirm whether the effect of bortezomib on autophagy has potentially relevant clinical implications, we assessed the *in vivo* effect of bortezomib on HCC xenograft tumors. Tumor-bearing mice were treated with vehicle or bortezomib or ΔBtz. Both drugs were injected intraperitoneally at 1.0 mg/kg twice a week for 2 weeks. All animals tolerated the treatments well without observable signs of toxicity and had stable body weights throughout the course of the study. No gross pathologic abnormalities were noted at necropsy. Our data indicated that bortezomib and ΔBtz showed similar effects on Huh-7 tumor growth. The size of tumor was reduced significantly after two weeks of treatment. To correlate biological response with the mechanism of action identified *in vitro*, the effect of bortezomib on autophagy in HCC tumors was examined. As shown in Fig. 5B and 5C, treatment of ΔBtz decreased the protein levels of CIP2A and P-Akt and significantly induced LC3 in Huh-7 cells similar to bortezomib. These data show that ΔBtz had significant anti-tumor effects *in vivo* and, importantly, suggest that bortezomib induces autophagy in HCC via a proteasome-independent mechanism.

## Discussion

This study reveals a novel mechanism by which bortezomib induces autophagy in HCC cells (i.e., the CIP2A-PP2A-Akt-4EBP1 pathway), and increases understanding of oncogenic proteins such as Ras, Akt and ERK that regulate autophagy [6], [41]. Interestingly, by using the compound ΔBtz that closely resembles bortezomib in chemical structure but lacks significant proteasome inhibitory ability, we demonstrated that this CIP2A-dependent regulation of bortezomib-induced autophagy is not necessarily dependent on proteasome inhibition. Bortezomib is presumed to inhibit the proteasome 20S core particle by the formation of a stable tetrahedral boronic acid intermediate with the N-terminal threonine residue of the active β-subunits of the 20S core particle [7]. Based on the importance of boronic acid functional group in blockade of the proteasome, we replaced bortezomib with ΔBtz that differs from bortezomib in its boronate group (Figure 4A). ΔBtz indeed showed little proteasome inhibition but nonetheless elicited CIP2A-dependent autophagy in a similar manner to bortezomib (Figure 4). This proteasome-independent CIP2A inhibition and autophagy mediated by bortezomib was further supported by the finding that other proteasome inhibitors (MG132 and lactacystin), did not significantly affect autophagy and CIP2A (Fig. 4D *left*) despite showing significant proteasome inhibition activity (Fig. 4D *right*). These data clearly indicate that the effect of bortezomib on autophagy can be independent of proteasome inhibition. Notably, the response of each cell line to the induction of LC3-I/LC3II by bortezomib was different (Fig. 1A) and was not identical to the according changes in CIP2A and P-Akt (Fig. 2A). One possible explanation is that these differences may be associated with distinct genetic backgrounds between cell lines. However, it is still possible that new targets or pathways (other than CIP2A/Akt pathway) might also play a role in mediating the effect of bortezomib on autophagy.

The proteasome and the autophagy-lysosome pathways are considered the two main routes for eukaryotic intracellular protein clearance and their linkage and interactions have been recent subjects of interest [42], [43], [44]. It is considered that these two cellular degradation systems are functionally coupled and suppression of proteasome pathway activates autophagy through induced ER stress [43], [45]. In this context, activation of autophagy functions to compensate for the impaired degradation of ubiquitinated protein induced by proteasome inhibitors and alleviates ER stress [14], [45]. Consequently, autophagy can protect cells from the toxicity of proteasome inhibitors. On the other hand, inhibition of autophagy can compromise degradation of ubiquitin-proteasome pathway substrates [46]. Evidence has shown that a combination of autophagy inhibitors and proteasome inhibitors promote cell death through both apoptosis and necrosis [14]. With our HCC model, we have shown that bortezomib induced autophagy through a proteasome-independent mechanism. Previously we confirmed that bortezomib can induce apoptosis through a CIP2A-PP2A-p-Akt mechanism, which is also dissociated from proteasome inhibition by bortezomib [40]. We demonstrated that bortezomib induced autophagy in HCC cell lines (i.e., Sk-Hep1, Hep3B, and Huh-7) that are also sensitive to bortezomib-induced apoptosis. Using our HCC model it is evident that through inhibition of CIP2A bortezomib elicits both apoptosis and autophagy, both of which are independent of proteasome inhibition. Furthermore, the evidence that CIP2A plays a major role in mediating bortezomib induced apoptosis and autophagy in HCC cells suggests that CIP2A may be a potential new drug target in HCC and that compounds/drugs that act as CIP2A inhibitors may have therapeutic potential in HCC. Whether currently known autophagy inhibitors such as bafilomycin A1, 3-MA or chloraquine can synergize with these CIP2A-inhibiting agents to kill cancer cells warrants future studies.

In addition to the finding that oncogenic CIP2A mediates bortezomib induced autophagy, this study identified p-4EBP-1 as a participant in bortezomib induced autophagy downstream of p-Akt. The role of p-4EBP1 was validated by ectopic expression of 4EBP1, which resulted in an increase in p-4EBP1, and subsequently reduced the effect of bortezomib on autophagy (Figure 3B). 4EBP-1 is known as downstream effector of PI3K/Akt/mTOR signaling that in non-phosphorylated form binds tightly to eIF4E and inhibits a key step in translation initiation [47], [48]. EIF4E is a key factor in cap-dependent translation initiation (including several important oncoproteins such as c-Myc, cyclin D1, hypoxia-inducible factor 1, and Mcl-1) that controls tumor cell growth and survival. Our data suggest that bortezomib downregulated CIP2A-PP2A-p-Akt associated p-4EBP1. It is possible that downregulation of p-4EBP1 suppressed eIF4E-dependent translation thereby promoting bortezomib-induced autophagy. This supposition is supported by a recent study showing that suppression of 4EBP1 expression resulted in re-sensitization of MYC-expressing prostate cancer cells to rapamycin-induced autophagy [49]. Indeed, we also noticed that bortezomib also suppressed the expression of 4EBP-1 in Sk-Hep1, and Hep3B cells (Figure 2A), which might also contribute to bortezomib-induced autophagy. Alternatively, PP2A may directly dephosphorylate eIF4E and contribute to bortezomib-induced autophagy [50]. Nevertheless, the exact mechanism by which p-4EBP1 participates in autophagy activation in HCC remains to be elucidated and further study is necessary. Despite the current results, the detailed mechanism by which bortezomib inhibits CIP2A remains unknown and further mechanistic studies are needed. The possible mechanisms through which bortezomib may affect the transcription of CIP2A include direct or indirect promoter regulation of CIP2A mRNA, epigenetic regulation of the *CIP2A* gene by DNA methylation or micro-RNA machinery, or affecting other as yet unknown molecules that may regulate CIP2A expression.

In conclusion, bortezomib induces autophagy in HCC cells through a novel proteasome-independent mechanism: CIP2A-dependent p-4EBP-1 downregulation. This study identifies the novel oncoprotein CIP2A as a major mediator of HCC cells to bortezomib-induced autophagy and suggests that CIP2A may be a potential new drug target in HCC. Furthermore, discovery of compounds/drugs acting as CIP2A inhibitors may have therapeutic potential in HCC therapy.
